# Entropic Analysis of Votes Expressed in Italian Elections between 1948 and 2018

**DOI:** 10.3390/e22050523

**Published:** 2020-05-04

**Authors:** Stefano Marmani, Valerio Ficcadenti, Parmjit Kaur, Gurjeet Dhesi

**Affiliations:** 1 Business School, London South Bank University, 103 Borough Road, London SE1 0AA, UK; marmanis@lsbu.ac.uk (S.M.); dhesig@lsbu.ac.uk (G.D.); 2 Leicester Castle Business School, De Montfort University, Leicester LE1 9BH, UK; pkcor@dmu.ac.uk

**Keywords:** Italian elections, politics, b-ary entropy, Gini index, Kolmogorov–Smirnov test

## Abstract

In Italy, the elections occur often, indeed almost every year the citizens are involved in a democratic choice for deciding leaders of different administrative entities. Sometimes the citizens are called to vote for filling more than one office in more than one administrative body. This phenomenon has occurred 35 times after 1948; it creates the peculiar condition of having the same sample of people expressing decisions on political bases at the same time. Therefore, the Italian contemporaneous ballots constitute the occasion to measure coherence and chaos in the way of expressing political opinion. In this paper, we address all the Italian elections that occurred between 1948 and 2018. We collect the number of votes per party at each administrative level and we treat each election as a manifestation of a complex system. Then, we use the Shannon entropy and the Gini Index to study the degree of disorder manifested during different types of elections at the municipality level. A particular focus is devoted to the contemporaneous elections. Such cases implicate different disorder dynamics in the contemporaneous ballots, when different administrative level are involved. Furthermore, some features that characterize different entropic regimes have emerged.

## 1. Introduction

**Figure 1:**
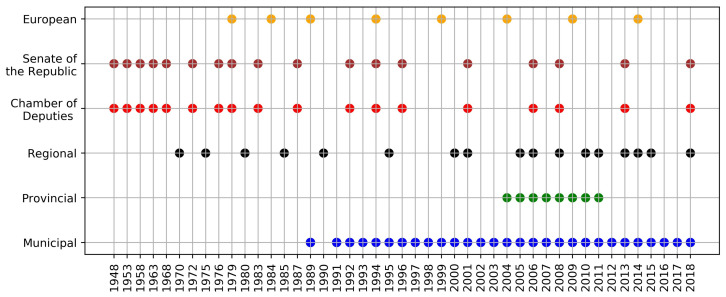
Each marker represents the type of election that occurred at least once in one municipality over the years present in our database. The colours are there just to help the reader.

The Italian Ministry of the Interior publishes the electoral data immediately after each Italian election round. The official available data proves the high number of voting turns that occurred over the years; Figure 1 provides an idea of the voting frequencies considered at all Italian administrative levels: Municipal, Provincial, Regional, National (Chamber of Deputies and Senate of the Republic) and European (the second turns are excluded). In many cases (35 specifically), the citizens have voted contemporaneously for electing people in different offices. We will refer to such phenomena as overlapping cases hereafter. Contemporaneous elections are peculiar cases that make possible the study of elections’ outcomes when citizens can make more than one choice to elect different offices at the same time.

This paper aims to use an alternative approach to study the citizens’ choices. To do so, we consider the various Italian elections’ outcomes as the realizations of a *Disordered System’s* states and we address the degree of disorder overcoming the assumptions often used in the voters’ behaviours studies. To run such a measurement we employ the b-ary entropy (or Tsallis’ entropy, see [1,2,3] for a wide review of the measure’s properties) on the votes’ distributions, then we use the Gini coefficient to further disentangle the data. In fact, given the usage of a non-extensive entropy formulation, an additional exploration has been performed. As a first step, to outline the advantages of using a free from assumptions measure as b-ary entropy is, we review some literature concerning the voters’ choices models and their main assumptions.

The *Split-ticket voting* behaviour, namely the tendency of the voters in selecting different parties or candidates according to the offices to be filled with democratic elections, is a tendency often detected (see [4]) when there are synchronized (overlapping) elections. The *vertical ticket-splitting* can be observed [5] as well when concurrent elections are studied (horizontal ticket splitting can occur when more than one equivalent roles have to be filled). The opposite of this behaviour is known as the *straight-ticket voting* (for the U.S. case see [6]); namely, the votes are expressed consistently in favour of the same party (or for the candidates expressed by a party), regardless the offices to be filled. In the context of this paper, we mention voters’ attitudes as examples of factors that could influence the electoral results, and therefore, the chaos in votes’ distributions, which is the core of interest in this paper.

Before stepping into a review of studies which modelled voter choices, it is appropriate to refer to [7]. It is a political study of the remarkable changes that occurred in Italy in 1996 when the left side coalition (namely, the members of Parliament who sits to the left of the President of the legislative assembly) won the national election after eventful years (from the famous *Tangentopoli* in 1992 to the beginning of the first Berlusconi’s government, 1994). In the 1996’s ballot, the so-called *Legge Mattarella* was applied for the first time, it divided the parliamentary seats by different type of electoral systems (mixed system), 75% of the seats were distributed through a majoritarian rule, while the remaining 25% was distributed through the proportional system (with minor corrections). The authors of [7] provide an idea of the needs of Italian parties in changing the electoral laws as well as the citizen responses. Furthermore, from such a paper, it is possible to grasp how tormented the modern Italian political history was. Crisis, conflicts and sometimes terrorism have characterized many years of Italian history, see [8]. In such periods, the complexity of the information processed by voters to make an informed decision in a democratic competition has been huge. Consequently, the attempts to model the voters’ behaviour is challenging and might lead to “divergent conclusions” as said in [4]. The amount of factors that influence the decision-making process of the voters is so large, that can be considered as the main source of disorder. For this reason, it is central to investigate the decisions with a free from assumptions view, looking at the realizations as pure manifestations of a chaotic framework, for figuring out realized paths and to perform an inductive study. Under this perspective, we present some salient studies to walk through some variables that contribute to characterize the disorder of the votes distributions.

The authors of [9] present an investigation of the *split-ticket voting* phenomenon during the 1998’s German Bundestag election. The authors employ logistic regressions and national surveys to test some behavioural voters assumptions specific for the German case due to its peculiar electoral system. In short, they conclude that voters tend to express preferences for parties just when they have an idea about the coalition to be voted, then preferring the parties with the highest chance to elect a candidate in the district of interest.

In [10], the authors report the determinants of a “dishonest vote”, namely “a vote casting in favour of a candidate who is perceived as dishonest by the voter herself” by using data from the *Second Italian Republic (2001–2008)*. To do so, they used survey data collected in that period. The authors concluded that “voting for a dishonest candidate depends only to a limited extent on the social characteristics and pre-existing political orientations of voters”, but it is grounded on two strong and simple voters’ beliefs “none of the candidates is an honest person, and/or that they are all alike on the whole” which do not allow the citizens to appreciate differences between competitors. The authors demonstrated that “the propensity to cast a dishonest vote is much higher among less civic-minded voters”, defining a feature to look for territorial clusters.

The authors of [11] have proposed a way to model the voters’ behaviour when contemporaneous elections have taken place. They investigated the voters’ choices when they contemporaneously express preferences for offices at different administrative levels, specifically they focused on the 2009 Italian election (Municipality and European ballots). The authors employed a variant of the Brown and Payne model [12] and they found some determinants of the vote splitting phenomenon specifically for the analyzed case of Terni, a medium-sized Italian city, which restored the Mayor in 2009. The authors describe the strong Brown and Payne model’s assumptions, and they come up with a new version of it, fairly representing the degree of uncertainty of their conclusions given the model peculiarities (e.g., the assumptions about the voters’ transaction matrix, especially when data at the polling station level is not available, see [13]). In [14], less invasive assumptions are involved thanks to the usage of the maximum entropy method (see [15] for checking out some of its features) to estimate the voters’ transition probabilities. The author defines the “maximum entropy” method as “a statistical framework that utilizes a concept of uncertainty based on information theory” and such a method does not require particular distributional assumptions (see [16] for an extensive presentation of the methodology).

The voters’ decisions for overlapping ballots are under investigation by many researchers. The key assumptions related to votes distributions for estimating the transition probability of preferences cannot be completely avoided due to the intrinsic complexity and the numerous variables involved. Some studies have eluded such a problem using aggregated data jointly with other types of information (e.g., metadata or survey) to test behavioural assumptions under different lights. In such cases, the new information taken into account is partial because the voters’ preferences and embedded determinants are secret. In addition, the degree of sincere disclosure depends on the interviewed voters will. The huge number of studies dealing with the voters’ choices determinants constitutes a proxy of the research interest for the topic as well as a measure of the complexity implicated by the variables. The substantiated importance justifies our research, which is designed to capture the relationship between elections outcomes and voters’ choices distributions overcoming the micro-structure of the voters’ decision-making elements.

In our analysis, we do not use ecological inference approaches [17] and so we avoid the assumptions connected to them because we do not aspire to conclude on the features of voters’ behaviours. We are not addressing the determinant of votes’ transitions at voters level. Instead, we move our steps from the observed citizens’ voting decisions thanks to the electoral aggregated data. In doing so, we do not consider the single choices to connect results with entropy. For similar reasons, we do not follow the steps of [12], which is an improvement of [17], but it still requires relevant assumptions on citizens’ choices to be able to model their selection processes. Further attempts of improving this family of methods have been produced, for example, in [11] and [18] but again, the key assumptions have not been overcome in modelling the citizens’ decisions. Such assumptions cannot be easily overcome in the agent-based model as well, see [19].

We focus on overlapping elections, therefore, the transaction probabilities needed to find the voters criteria cannot be easily determined unless other information (like that coming from surveys) are taken into consideration. Furthermore, the quasi-perfect contemporaneous moment in which the votes are expressed leads to prefer observing the chaos manifested via the preferences expressions from the citizens, without considering all the rest. Our analysis is devoted to the differences in disorder generated from the citizens’ final choices; namely, we examine the state of the system after their decision-making processes have taken place.

Italy has numerous overlapping elections, for example, it had six Regional and Municipal ballots that occurred on the same day, see Table 1. Here, we are observing and studying the consequences on distributions of whatever is the strategies employed by voters (for example the tactical “split-ticket” phenomenon, see [20,21,22]. The dataset we employ covers 70 years, for a number of 163 elections. The votes per each party in competition, for each type of election and for each date have been aggregated and stored at the municipality level. Therefore, for each ballot type (European, Senate of the Republic and Chamber of Deputies, Regional, Provincial or Municipal) we have the number of votes obtained by the competitors as well.

To investigate the electoral outcomes’ distributions when many different parties are involved, we perform the analysis using the *b-ary entropy* [23,24,25] and with the Gini coefficient [26] we further confirm the findings. The former is appropriate to capture the votes distributional features and degree of disorder at the municipality level across the years. Furthermore, because b-ary entropy is a normalized measure of the chaos found within a discrete distribution, it is a non-extensive quantity that allows comparison of election outcomes with different numbers of candidates across municipalities and elections (for example, see [27] where the additive problem is treated). Anyway, in employing such a measure, we are in line with empirical entropy applications like [28]. We calculate the Gini Index, which is particularly suitable to validate the entropic analysis and to provide a different view of the distributional votes’ distributions across parties. It measures the polarization of the distributions and, therefore, provides a different indication of the distance from a uniform distribution. The Gini index is the ratio of the area that lies between the equality line and the Lorenz curve over the total area under the equality line.

The b-ary entropy and the Gini index are computed for each municipality on each election date and for each type of ballot, namely by using the number of votes obtained by each party that has competed. In this way, each municipality has entropy and a Gini value per election and they are comprised between 0 and 1. Specifically, if the voters have expressed all their preferences for a single party in a municipality, then the entropy will be 0 and the Gini index will be 1; on the other hand, a homogeneous division of the votes between the parties would lead to the entropy equal to 1 and Gini equal to 0. Anyway, for entropy and Gini, some considerations on the ranges are reported in Section 3.

The concept of entropy has been employed in different fields with similar meanings, for example, see [29,30,31,32], where the authors have applied it in the financial field, [33] where the authors presented a case study of complex information measurement with entropy, [34] for an excellent example of entropy employed to investigate the complexity or diversity of 995 music groups and [35] for a measure of reliability connected to the concept of entropy.

Scholars have differently employed entropy in political studies, for example in [36], the authors have investigated the voters’ behaviours thanks to surveys filled by Belgian citizens via non-partisan and partisan profiles websites (it consists of on-line quizzes to profile the users from a political point of view). The Shannon Entropy has been employed to assess if the voters’ opinions gradually converge or crystallize around their preferred party during the weeks before the elections.

In [37], the entropy helps in testing the hypothesis that the “Contract with America” reduced the voters’ uncertainty in 2004. Specifically, the authors wanted to evaluate how voters interpret different levels of information about Republican House candidates in the US during the mid-term election. Unfortunately, as it is common in this type of study, the researchers have met problems with non-observable variables. So they overcame the problem applying the entropy to data coming from surveys in combination with some assumptions [36,37] focus on analyzing the voters’ decision-making process. In our work instead, we look at their choice directly; after that, all the variables have expressed their weights on the citizens’ decisions. Speaking about direct observations [38], analysed election turnout in many countries using the Shannon entropy to study the voters’ conformity behaviour. While [39] have investigated the proportions of abstentions, blank and null voting papers, and valid votes via the Shannon entropy. Their approach is similar to ours, their data is aggregated at the municipality level for 11 countries while we focus on the Italian case only, but differently from [39] we cover a wider time. Concluding, in [28], the authors have studied the relationship between entropies as a measure of citizens dissatisfaction and abstentions in 15 European countries.

The Gini index has an enormous list of applications as well; the main one is the measurement of income inequality from which the Gini Index is born in [26] (see for example [40], a study of inequalities in Canada). However, it has been applied also to study the economical characteristics of certain classes of Italian cities in [41], recently, to improve the performance of clustering algorithms [42] or for measuring inequality in water usage [43]. On the relationship between Gini and b-ary entropy, it is worth to mention [44,45] where the Gini and Tsallis’ entropy are used to model income inequalities or, as in [46], to improve the performance of decision trees classifiers in the field of machine learning.

At the best of our knowledge, this paper is the first one dealing with such a wide database regarding Italian elections. Furthermore, we are unique in using the b-ary entropy and the Gini coefficient to measure the level of disorder in the Italian elections with a specific focus on the days during which multiple choices have been expressed by the citizens. In doing so, we consider the concurrent elections as complex system’s manifestations and, via the entropy distributions we address the similarities of its states.

The present study contains Section 2 where the database is presented, Section 3 to describe the utilization of the entropy and Gini Index, and finally, Section 4 and Section 5 to show the results and comment on them.

## 2. The Italian Elections Database

The dataset used is one of the largest and most complete that can be found about Italian elections. It has been provided by the Italian Ministry of Interior.

The raw original database has been converted in a SQL version. During the transformation, it has been organized in subdivisions. The type of election is used to create the first division. Namely, we have six categories: Municipal, Provincial, Regional, Chamber Of Deputies, Senate Of The Republic and European. The Municipal and Provincial cases contained the second ballots which have been removed in this study (the Italian law no. 81 of 1993 introduces the direct election of the mayor, the president of the province and the municipal and provincial councils, and a second-round ballot when no candidate has obtained more than 50% of the votes). The second subdivision divides the database into two sections per each election. A part describes the competitors (parties names and details of the single candidates) the other describes the results obtained from the competitors (votes collected by parties and single candidates). The original framework of the data had geographical references just through the territorial segmentation designed by the different electoral laws (e.g., electoral constituency for the Chamber of Deputies and Senate of the Republic). We have organized the database by using the municipalities as references in order to get back a political geography view of the information. On this step, we have met some difficulties because the municipality names are the unique label useful to realize such a scope. Sometimes the municipalities have been subjected to changes like merging between entities because of the low population or expense rationalizations (e.g., Brescello–Viadana, which is born by the merging of Brescello and Viadana). Because of these facts, the total number of municipality varies from election to election, creating a different list of labels per date.

The time covered goes from April 1948 to October 2018. In this period, we had 163 number of days in which at least one municipality was involved in the democratic process for filling offices. In each one of these dates, the electoral results of the parties competing have been pulled and used to calculate the b-ary entropy and the Gini Index. Therefore, at the municipality level, the entropic behaviours are systematically captured, as described in the next section.

In 35 days, the offices elected belonged to different administrative entities originating multiple choices for the citizens. Most of the times they have expressed two choices, but in some cases, the synchronized elections have involved more than two administrative levels, like in April 2008, where Municipal, Provincial and National elections have occurred at once (in these cases, the media and the politicians have defined such events as “Election Day”).

The final database contains the entropy levels and the Gini index for each Italian municipality across the years.

## 3. Methodology

We move from the votes of each party during the elections that occurred in Italy before October 2018. Specifically, we have collected data regarding *N* elections called for European, National (both the Chamber of Deputies and Senate of the Republic), Regional, Provincial and Municipal offices. In each ballot, $M_{n}^{i}$ rivals (now on referred as lists to intend lists of candidates or parties) have competed, where i=1,...,D, with *D* representing the number of municipalities for which the data is available in the *n*-th electoral turn. Therefore, it is possible to access the number of votes per list in each municipality, getting election outcomes per area. The number of lists competing in the local elections $M_{n}^{i}$ varies from district to district, while the Nationals have a (more stable) number of list per zone. The reason to get different lists even when the National elections run may vary from case to case; one of the most relevant facts is of a political type. Indeed, depending on the electoral law active from time to time (Italy has been proactive in adopting and making electoral systems), the parties had to collect a certain number of signatures from citizens at regional or electoral constituency level to be able to join the democratic contest. If a party was not able to collect enough citizens’ signatures to meet the mandatory threshold in an area, then it could not compete for that ballot.

Regardless of the election type, we computed the b-ary entropy [23,24,47] on the results obtained at municipality level according to the following formula:

(1) $$H_{n}^{i}=-\sum_{j=1}^{M_{n}^{i}}p_{j}\log_{M_{n}^{i}}p_{j}\;\forall \;i\in E_{n};n\in N$$

where $H_{n}^{i}$ is the entropy based on the election outcome got in the municipality *i* during the *n*-th round of voting ($E_{n}$ is the set of municipalities and *N* is the set of elections), $p_{j}$ is the share of votes obtained by the competitor *j* over the total votes expressed in that municipality and $M_{n}^{i}$ represents the number of parties competing in that city (the number of parties may vary across municipalities even during national elections for example. It may happen for various political reasons briefly examined in Section 4). In considering the shares of votes in this fashion, we are in line with studies like [48], where the same has been done for the Lithuanian parliamentary elections. However, differently from that paper, we have considered all the parties’ results, while in [48], the author has considered just the parties that overcame the electoral barrier sometimes imposed by the law.

The b-ary entropy can be seen as a standardization of the Shannon entropy, which ranges between 0 and $\log_{2}M_{n}^{i}$. The following relationship holds:

(2) $$0\leq \frac{-\sum_{j=1}^{M_{n}^{i}}p_{j}\log_{2}p_{j}}{\log_{2}M_{n}^{i}}\leq 1\;\forall \;i\in E_{n};\;n\in N$$

Therefore, applying a change in logarithms’ bases one gets:

(3) $$0\leq -\sum_{j=1}^{M_{n}^{i}}p_{j}\log_{M_{n}^{i}}p_{j}\leq 1\;\forall \;i\in E_{n};\;n\in N$$

The absence of chaos is identified with 0, namely, all the votes have been expressed for a single party, on the other hand, 1 stands for a homogeneous distribution of votes between adversaries. In applying this standardization, we have met the non-extensivity problem of this measure, even if it has been used in a wide range of interdisciplinary fields [49].

With the same logic, and to disentangle the problem, the Gini coefficient [26] (sometimes quoted as Gini hereafter) has been employed, but some considerations are needed. The standard formalization of the Gini is the following:

(4) $$G_{i}^{n}=\frac{\sum_{j=1}^{M_{i}^{n}}\sum_{h=1}^{M_{i}^{n}}|x_{j}-x_{h}|}{2M_{i}^{n}\sum_{j=1}^{M_{i}^{n}}x_{j}}\;\forall \;i\in E_{n};\;n\in N$$

where, *i* indicates the *i*-th municipality involved in the *n*-th round of voting ($E_{n}$ is the set of municipalities and *N* is the set of elections), $x_{j}$ and $x_{h}$ are the number of votes obtained by the lists *j* or *h*, respectively, and $M_{n}^{i}$ is the number of parties competing during such a ballot. Therefore, $G_{i}^{n}$ is the value of the Gini Index for the municipality *i* at the *n*-th election. If all the analysed observations are positive and the targeted dataset is large enough, the following condition holds:

(5) $$0\leq \frac{\sum_{j=1}^{M_{i}^{n}}\sum_{h=1}^{M_{i}^{n}}|x_{j}-x_{h}|}{2M_{i}^{n}\sum_{j=1}^{M_{i}^{n}}x_{j}}\leq 1\;\forall \;i\in E_{n};\;n\in N$$

The Gini coefficient presents different drawbacks depending on the type of data analysed. For example, if the observations considered contain some negative numbers, there is the need of a re-normalization because a value greater than 1 could be met (see [50,51]) or, in small samples, the Gini is likely to be biased downward [52]. The latter case needs to be addressed because it is present in our study. Indeed, the value of the Gini for an election with two parties can be less than one-half; specifically, in the extreme case of a party that gets 0 votes and the other gets all the votes, the Gini results to be 0.5. As estimated in [52], “The Gini exhibits a bias of up to 7.5% even for a sample size of 20.” To fully correct such a bias there is the need for considering additional complexity coming from the distributions of the data. Anyway, for comparison purposes and so for assessing the results of the entropic analysis, we neutralize such a bias as much as possible, normalizing the upper bound of the Gini for each municipality, election type and turn. Namely, we have generated the synthetics cases in which the various elections have been won by a single party. Furthermore, we have assumed that such a party collected all the votes in each municipality, election type and turn. Then, we have calculated the Gini for such cases. These numbers are the Gini upper bounds here identified as $U_{i}^{n}$. For example, if in a Municipality, three parties have competed and they got the following numbers: party A, 10 votes, party B, 20 votes and party C, 70 votes, the Gini upper bound has been calculated on the following hypothetical outcome: party A, 0 votes, party B, 0 votes and party C, 100 votes. Consequently, Equation (5) becomes:

(6) $$0\leq G_{i}^{n}\leq U_{i}^{n}\;\forall \;i\in E_{n};\;n\in N$$

which is equivalent to write:

(7) $$0\leq \frac{G_{i}^{n}}{U_{i}^{n}}\leq 1\;\forall \;i\in E_{n};\;n\in N$$

Concluding, we have compared the following adjusted Gini with the b-ary entropy.

(8) $${G_{adj}}_{i}^{n}=\frac{G_{i}^{n}}{U_{i}^{n}}\;\forall \;i\in E_{n};\;n\in N$$

Once we get the entropy and the adjusted Gini for each municipality, for each democratic call, we compare the entropy empirical distributions realized in different elections between each other and then we do the same for the Gini empirical distributions. After that, utilizing Pearson’s and Kendall’s correlations, we can assess the relationship between the two measures to validate the findings.

The main objective is to study the contemporaneous ballots pairwise (e.g., Regional election with European election) to capture information about the outcome of voters’ decisions in an aggregate fashion. In this way, we look at voters’ choices under a different perspective, particularly extracting information from the distributional features of the manifested chaos in each district. To compare the distributions between each other (not mixing entropy and Gini), we employed the Kolmogorov–Smirnov test (see an illustrative example in [53] or [54]) between the resulting figures in concomitant ballots. Table 1 contains the type of election that happened at the same time and the number of municipalities for which the results have been reported. The case of Chamber of Deputies and Senate of the Republic presents some differences in the number of municipalities involved due to minor data inconsistencies. We have run the analysis where the number of the data points is large enough to allow for the presence of a meaningful cumulative distribution function, as the Kolmogorov–Smirnov test (KS test from now on) is based on it. For this reason, we have not considered the 17/11/2013 and 17/04/2005 elections.

Sometimes, the contemporaneous rounds of voting involved just subsets of zones of Italy (e.g., the case of 25/05/2014, where Piemonte and Abruzzo renewed their regional offices), therefore, running the KS test on the overall data requires a further check to exclude spatial effects. To pursue such a scope, we have run the test a second time but selecting just the municipalities for which the data is present in all the elections that occurred contemporaneously (a set intersection on the municipalities’ names). For example, for the case of April 2000, we have selected the municipalities present for the Regional elections as well as for the municipal ones and we have compared their entropies via the KS test. Therefore, we have two KS test statistics, one for the entropy comparison and another for the Gini comparison.

## 4. Results and Comments

In this section, we report the result of the entropic and inequality analysis performed on the Italian elections between 1948 and 2018. The section is divided into four parts, the first two where we present the general dynamics of the b-ary entropy and the Gini coefficient along the years for the majority of the ballots and the last two in which the results for the contemporaneous elections are reported.

### 4.1. The b-Ary Entropy of the Italian Elections

A summary of the entropy calculated with the election results coming from Italian municipalities for each ballot in the dataset is reported in Table 4. To better summarize the outcomes, we have divided the time into three chunks.

Figures 4–6 are helpful to clarify the changes in entropy that occurred along the years for the European elections, Chamber of Deputies and Senate of the Republic. The other types of democratic calls are not suitable for such a representation because on certain dates they have involved just a part of the country.

The maximum entropies for the Municipal elections are recorded in northern towns characterized by low population. All of them reached in recent elections (after 1995).

For the Provincial cases, we have the three highest entropies that occurred in the Veneto region, namely Domegge di Cadore, La Valle Agordina and Sedico, with respectively 0.957, 0.942 and 0.934. If one extends the analysis to the top ten entropy levels, it is possible to notice that they also fall into northern regions, particularly in towns characterized by low population. However, most of them were recorded during the 2004 election.

On the other hand, for the municipality with the minimum level of entropy, one cannot see a particular trend apart from noticing that most of them occurred in 2009 and 2004 ballots.

The Regional elections manifest a maximum in Campobasso on 16/10/2011 with 0.95. In descending order, the following are Pietrabbondante (Molise), Cugnoli (Abruzzo) and Sant’Agapito (Molise). All of them have a low population and they got, respectively, 0.948, 0.944 and 0.942 on 23/04/1995. Extending the analysis to the most entropic municipalities, one finds mostly town and villages belonging to the Molise region.

The minimum entropic municipality in the Regional ballots is Sizzano with zero entropy obtained on 16/04/2000. It is quite an interesting case because Sizzano had 1266 potential voters, 972 of them showed up at the cabinets and all of them spoiled the votes (expressing invalid preferences) or left voting papers white. To find some ideas for justifying such a behaviour, see [55]. The authors documented the relationship between the margin of victory of the leading candidate over the nearest rival and the share of invalid ballots; they justify their findings writing “we then show that this relationship is unlikely to be driven by voter mistakes, protest, or electoral fraud. The explanation that garners most support is that election officers and party representatives rationally allocate more effort in detecting invalid ballots when the stakes are higher, i.e., when the electoral race in the district is closer.” The authors’ focus falls on the Italian parliamentary elections, but if their results might be extended, surely this is an interesting case (outlier) to comment.

The lowest entropic municipalities just before Sizzano are Castelpizzuto with 0.095 on 8/06/1980, Morterone with 0.11 on 8/6/1980 and, finally, Aquila di Arroscia with 0.116 on 7/6/1970. They all referred to quite old ballots.

Talking about the Italian parliamentary elections (Chamber of Deputies), the three highest positions for their entropy levels are held by Saint-Pierre, Cogne and Saint-Denis (all from Valle d’Aosta region) with one obtained on 19/05/1968. Interestingly, all the top 10 positions are held by towns from Valle d’Aosta, which got their outcomes on 19/05/1968 or 28/4/1963.

The Chamber of Deputies ballot has zero as the minimum level of entropy. It is reached by Valsavarenche (none went to vote, the population was made of about 200 people) and Massa di Somma on 14/06/1987 (no data available), Staiti (there were three voters that spoiled the ballot papers on 21/04/1996), Santa Maria la Carità (there is no data for 3/06/1979) and Spera, where all the 303 valid votes have been expressed in favour of “Democrazia Cristiana” on 18/04/1948.

The Senate of the Republic ballots present a maximum entropy in Saint-Pierre with one obtained in 1968. It is followed by other towns from the Italian’s northern regions (Friuli Venezia Giulia, and Toscana). These municipalities got high levels of entropies (around one) during the 1948, 1968 and 1996 elections.

The Municipality with the maximum entropy for the European election is Salza di Pinerolo 13/06/1999 with 0.93. Then Ribordone with 0.899 in 13/06/1999 followed by Noasca and Issiglio with 0.897 and 0.894, respectively, all of them on 13/06/1999. It is interesting to notice that all of them belong to the Piemonte region and they have a population below 500 people. The lowest entropy level for the European election occurs in the Trentino Alto Adige region, in a small town, during the 1979 and 1984 elections. The municipalities are Selva dei Molini with 0.035, Terento with 0.042 and Verano with 0.042 as well. All the first lowest level of entropies are held by municipalities of Trentino Alto Adige.

### 4.2. The Adjusted Gini Coefficient and the b-Ary Entropy in the Italian Elections

The summary of the results are presented in Table 4, columns G. Their comparison with the columns H can be consistently done jointly with a visual inspection of Figures 4–6. These graphs report the b-ary entropy (upper) and adjusted Gini (lower) along the years for the European elections, Chamber of Deputies and Senate of the Republic. As already mentioned, the other types of democratic calls are not suitable for representing the phenomenon along the years because certain ballots have partially involved the country.

To verify the validity of outcomes presented in Section 4.1 and to give strength to the Conclusions, we calculate the Kendall’s τ correlations between the b-ary entropy and the adjusted Gini.

(9) $$\tau^{n}=Corr_{\tau }\left(H^{n},{G_{adj}}^{n}\right)\;\forall \;n\in N$$

where $H^{n}$ and ${G_{adj}}^{n}$ are, respectively, the array of b-ary entropy and adjusted Gini index obtained for the *n*-th electoral turn.

**Figure 2:**
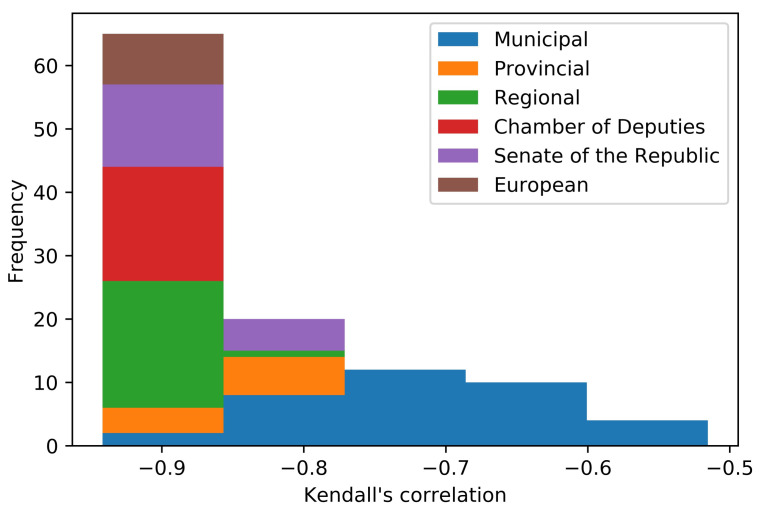
Kendall’s τ correlation between the b-ary entropy and the adjusted Gini coefficient for each ballot in the dataset. It has been calculated through the relationship (9) and it reports all the correlations given that all of them are statistically significant at 0.1%.

With τ, we measure the rank associations of the considered variables. A negative Kendall’s correlation signifies that as the rank of one variable is increased, the rank of the other variable is decreased. The correlations analysis returns all negative and statistically significant (at the 0.1% confidence level) τ. This validates all the results, and in Figure 2, a detailed view of the τ is reported.

### 4.3. The Entropy in the Contemporaneous Italian Eelections

To compare the entropy distributions calculated with parties’ performances at the municipality level, we have run a Kolmogorov–Smirnov test for each couple of contemporaneous elections. The compared ballots along the years are:

- European and Municipal (5 times);
- Chamber of Deputies and Senate of the Republic (18 times);
- Chamber of Deputies and Regional, Senate of the Republic with Regional (2 times each couple);
- Chamber of Deputies and Provincial, Senate of the Republic and Provincial (once per each couple);
- Chamber of Deputies and Municipal, Senate of the Republic and Municipal (2 times each couple);
- Regional and European (once), Regional and Provincial (2 times), Regional and Municipal (6 times);
- Provincial and European (2 times), Provincial and Municipal (9 times).

Every date in which there have been overlapping elections and the municipalities involved were less than 100 have been removed from the analysis (e.g., 17/04/2005 and 17/11/2013, see Table 1).

The most peculiar results are reported in Table 3. The KS test statistics are all very high, manifesting the different entropic behaviours for almost all the synchronised elections. These outcomes can be further confirmed by comparing the different stats resulting from Table 4. For each type of ballot that occurred on the same day, two different comparisons have been run to explore the spatial effect:

- *“Whole”*—where all the municipalities for which contemporaneous elections’ results are present in the database have been considered into the KS test.
- *“Intersection”*—where just the entropies of the municipalities common to both the type of the contemporaneous elections are considered in the KS test.

For example, in the recent ballot of 04/03/2018, when the *“Intersection”* is considered, the KS test is performed on the municipalities belonging to Lombardia and Lazio regions, selecting the same boroughs for both the elections types (Regional versus Chamber of Deputies or Regional versus Senate of the Republic). Such a distinction is particularly useful when the local elections (Regional, Provincial and Municipal) are compared to European or National. If one does not consider just the intersection, spatial effects/biases could be introduced. It is important to remark that in the *“Intersection”* comparisons, if the resulting number of common municipalities is inferior to 100, the KS test has not been performed. Anyway, in the paper, we report just the comparison for the interception.

The 10 highest and lowest KS statistics with their respective information in Table 3. It is interesting to notice the number of times local elections are involved in the highest KS statistics subtable. In most of the cases, the Municipal elections are involved introducing the main source of difference. Indeed, on the other hand, looking at the lowest KS statistics list (Table 3, second part), it is immediately clear that the closest distributions are those coming from the entropic behaviours of the Chamber of Deputies and Senate of Republic voters.

**Figure 3:**
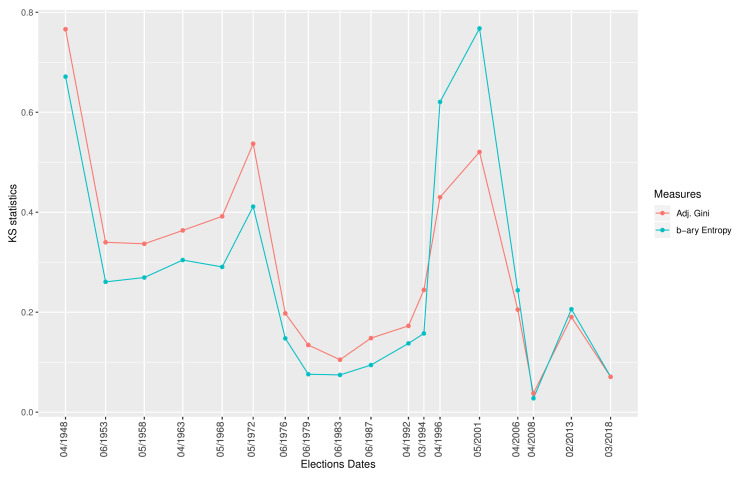
The Kolmogorov–Smirnov test (KS) test statistics, obtained by comparing the b-ary entropies distributions of election results for the Chamber of Deputies and the Senate of the Republic, are represented by the blue line, while the red line represents the same information when the adjusted Gini is used. The lower the level of the statistics, the more the distributions are unlikely to be similar.

The comparison between the Chamber of Deputies and Senate of Republic is the most common. Indeed, Italy has a bicameral system and the respective offices have always been filled thanks to democratic calls that occurred on the same days. Figure 3 shows the KS statistics coming from the comparison of the b-ary entropy distributions for the aforementioned type of elections. They originate from a pairwise test of the elections that occurred on the dates listed on the X-axis of the figure. The graph presents two regimes, one before and one after the famous corruption scandals *“Mani Pulite”* [56,57].

### 4.4. The Adjusted Gini and the b-Ary Entropy in the Contemporaneous Italian Elections

To compare the outcome coming from the two measures in each ballot, we have calculated the Pearson correlation between the b-ary entropy levels and the adjusted Gini in each election.

(10) $$\rho^{n}=Corr_{\rho }\left(H^{n},{G_{adj}}^{n}\right)\;\forall \;n\in N$$

where $H^{n}$ and ${G_{adj}}^{n}$ are, respectively, the array of b-ary entropy and adjusted Gini index obtained for the *n*-th electoral turn.

The correlation is statistically significant at the 5% of confidence level in almost all the cases (just 15 elections resulted in having a non statistically significant relationship when the entropy is compared with the Gini considering all the concurrent elections listed in Table 1). In Table 2, the non-significant cases are reported. They all occur for the municipal election.

The Kolmogorov–Smirnov test for each couple of contemporaneous elections have been calculated on the adjusted Gini coefficients. The considered elections are the same described in the previous section, namely those used for the b-ary comparison. Hence, for the cases in which less than 100 of municipalities are involved, the KS test is not run. Furthermore, the *“intersection”* is used. The results are in line with those presented in Table 3, namely the adjusted Gini distributions populating the top and the lowest 10 KS statistics table are the same, but the rankings within the lists are slightly different. The Municipal elections are involved in most of the cases with the highest KS statistics. While, in the lowest elections’ KS statistics, Chamber of Deputies and Senate of Republic are confirmed.

Figure 3 shows the KS statistics coming from the comparison of the b-ary entropies distributions and the adjusted Gini distributions for the Chamber of Deputies and Senate of Republic comparisons. The two lines in the graph are synchronised since the striking 1992, as discussed before.

## 5. Conclusions

This paper deals with one of the widest datasets of Italian elections. The b-ary entropy is used to carry out a measure of the disorder in voters’ choices at the municipality level. In using such a functional version of entropy we encounter the limitations belonging to a non-extensive quantity. On the other hand, it allows comparisons of election outcomes with different numbers of candidates, which is the main purpose here, especially for the contemporaneous elections. In this respect, the usage of Gini is a consistency validation of the findings when the conclusions on comparisons are grasped.

From a general point of view, the Italian elections present a quite high level of entropy, which has changed along the years under different perspectives. In this paper, we consider Municipal, Provincial and Regional elections as locals; see Figure 1 to assess their occurrences. The local offices have been renewed in different dates and, by the time passing, their synchronization gets lost, spreading the ballots along the years. From time to time, this involves part of the country in the democratic process for specific situations (e.g., early cessation of a Municipality board due to mafia infiltration). It makes difficult concluding on general entropic frameworks. However, it is possible to have an idea of the general disorder carefully commenting on the entropy results. Table 4 shows that the Municipal ballots have a slightly negative trend over the years; in fact, the mean values are decreasing and the standard deviation is quite stable. The decreasing kurtosis and the change in sign of the skewness helps in assuming a change in the concentration of the values. It looks like that in most of the municipal competitions the votes do not tend to be homogeneously distributed across the parties or that main players (maybe two) collect the majority of the votes and the rest goes to minor participants. The lowest average entropy between all the elections types has occurred in the municipal case. Certainly, a central role is played by the villages in which few participants have competed but one has collected almost all the votes, drastically lowering the entropy. One could hypothesize that the main change occurred due to the reform of the electoral law (1993), see [58,59]. Nevertheless, the adjusted Gini coefficient confirms these findings.

In Table 4, the Regional ballots have the highest average entropy with a maximum of 0.71 in the years between 1996 and 2018. Between 1948 and 1971, when the observations are about 6683, we have an average entropy of 0.64 very close to the median. Looking at the ratios μ/σ, we can say that the means are quite meaningful. Concluding, the regional elections entropies are more in line with the National or European rather than the other locals from a statistical point of view. The negative skewness and the lower kurtosis is a further sign of it because they are different from the other two. With a reverse logic due to the adjusted Gini nature, the results are confirmed as well.

**Figure 4:**
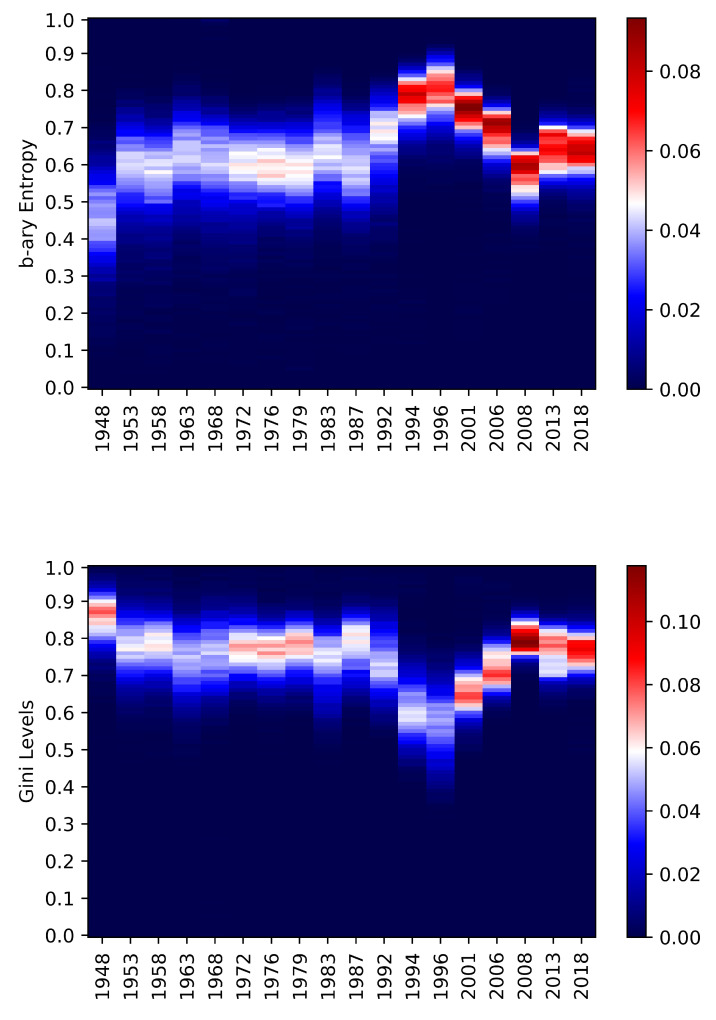
Chamber of Deputies—The heat-map represents the relative frequencies of the b-ary entropies (**above**) and the adjusted Gini (**below**) occurred on the dates in the *x* axis. The *y* axis represents the 100 bins obtained between the minimum and the maximum values obtained for the Chamber of Deputies type of elections. The colour represents the number of municipalities in that particular level of entropy/Gini (bin) for that date. It goes from blue (low frequency) to white (average frequency) to red (high frequency, most of the municipalities).

**Figure 5:**
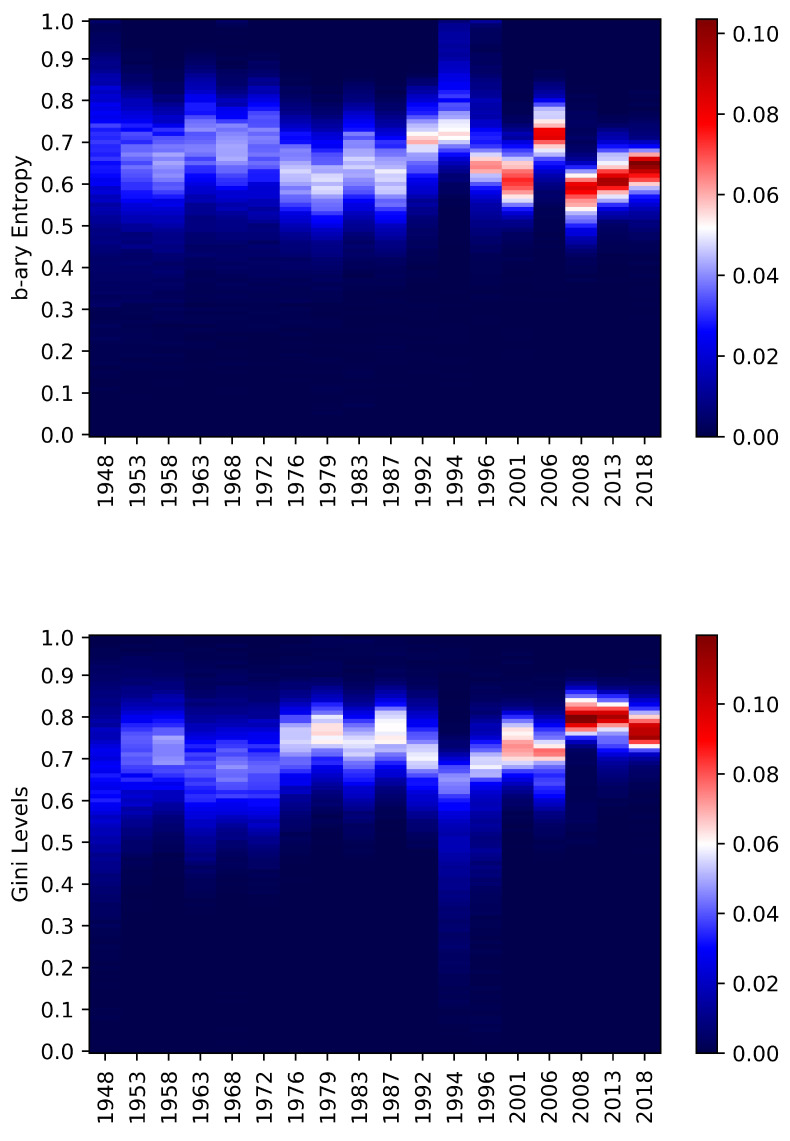
Senate of Republic—The heat-map represents the relative frequencies of the b-ary entropies (**above**) and the adjusted Gini (**below**) occurred on the dates in the *x* axis. The *y* axis represents the 100 bins obtained between the minimum and the maximum values obtained for the Senate of the Republic type of elections. The colour represents the number of municipalities in that particular level of entropy/Gini (bin) for that date. It goes from blue (low frequency) to white (average frequency) to red (high frequency, most of the municipalities).

Chamber of Deputies and Senate of the Republic have many common points from the entropic point of view; furthermore, they involved the entire country all the time. The average entropy levels are slightly dominant for the Chamber of Deputies just on the last time span while in the other cases, the Senate has been dominant. From a general perspective, the two chambers present quite synchronized increasing entropic behaviours. Figure 4 and Figure 5 are helpful to better understand the dynamics. In both cases, there is a change that occurred between 1992 and 1994. After that, the entropy distributions became more concentrated around the means. By looking at kurtosis and skewness of National elections entropies during the period 1996–2018 in Table 4, one can see further confirmations of change in the regime. These differences might be coming from tricky electoral laws, which tend to be so in Italy (see [60]) or from political crises that have influenced the voters’ choices. The adjusted Gini provides with a confirmation about these facts. The figures are synchronised and they make the situation more evident due to the construction of the coefficient. The lower graphs of Figure 4 and Figure 5 provide a clear visual intuition of this.

**Figure 6:**
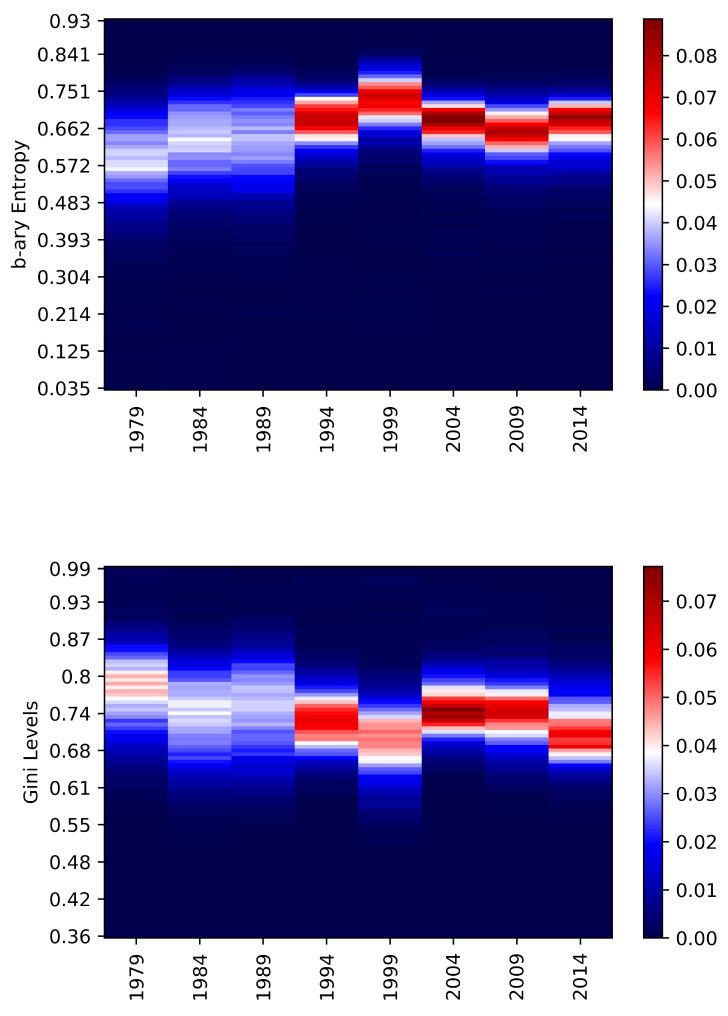
European elections—The heat-map represents the relative frequencies of the b-ary entropies (**above**) and the adjusted Gini (**below**) occurred on the dates in the *x* axis. The *y* axis represents the 100 bins obtained between the minimum and the maximum values obtained for the European type of elections. The colour represents the number of municipalities in that particular level of entropy/Gini (bin) for that date. It goes from blue (low frequency) to white (average frequency) to red (high frequency, most of the municipalities).

Finally, the European elections have increasing levels of average entropy and decreasing levels of adjusted Gini coefficient. The standard deviations slightly decrease, manifesting an increased concentration. There has been a change in regime in this case as well; indeed, Figure 6 shows a remarkable difference after 1989. A very important year for the European Union, for example, Spain and Portugal voted and after a few months, and there was the fall of the Berlin wall. The skewness and the kurtosis levels additionally prove it. The negative entropy skewness stays quite constant over the years while the kurtosis reaches the highest values of the table. In this case, the Gini analysis results provide similar hints apart for the fact that the highest kurtosis has been registered for the Senate of the Republic and the European elections.

The general picture shows a widespread increment in entropy across the country’s municipalities for all the elections apart from the municipal ones (the differences between National and local elections is a long-running research topic, see [61]). Furthermore, a general concentration around the average entropy levels has been met. The municipal elections behaviour can be a further sign of the relevance of spatial effects, while the concentration around the means can be explained with increased polarization in electoral campaigns. It is quite evident the different paths followed by the local and the national election. The latter tends to be more led by the general political discussion within the country. Therefore, the increased capacity of the parties in capturing the attention of the voters, so in making the electoral campaigns more effective, ended up contributing to new entropic dynamics put in place by the voters’ choices.

In addition, based on the figures presented in Section 4.1 and Section 4.2, we conclude about the presence of some natural clusters when addressing the features of the places/elections in which the highest–lowest entropies have been calculated.

- Municipal—The highest entropies are recorded in the northern Italian area, most of them in recent elections, namely between 1995 and 2016. The minimum entropy level is zero and many municipalities resulted with it. For example, San Didero, Bosio and Berzano di Tortona are very small villages where the citizens have voted for the unique party in competition. Most of the cases having zero entropy are like these. Others are affected by different voters behaviours, e.g., all made invalid their votes or data issues.
- Provincial—The highest levels of entropy have occurred in central-northern regions mainly during the 2009 and 2004 elections. The towns involved are all quite small apart from Bologna. The 10 municipalities with the lowest levels of entropy are small and they recorded such results during the 2009 and 2004 elections. This is due to the high variability within the country.
- Regional—Such cases present a different situation. The 10 highest entropies are obtained by municipalities of centre-south of Italy involved in the elections of 2011 and 1995. The lowest levels are again obtained from municipalities characterized by low population, the results of which have been mainly obtained in 1970 and 1980.
- Chamber of Deputies—The most entropic municipalities belong to Valle d’Aosta region and they are all very small. Their entropy values have been recorded during the 1968 and 1963 elections. There are six small municipalities with entropy equal to zero. They are peculiar cases of missing data, votes wrongly expressed or spoiled, as happened in the other cases.
- Senate of the Republic—The highest position is held by Saint-Pierre, which is the same town having the first position for the Chamber of Deputies. The other municipalities occupy the first 10 positions because their entropic figures come from the northern area. The elections involved are 1948, 1968 and 1996.
- European—The most entropic municipalities are tiny and they belong to the Piemonte region. Their electoral outcomes come from the 1999 election. The 10 municipalities with the lowest entropies come from the north of Italy, specifically from Trentino Alto Adige region. The towns in this case are slightly bigger and their results have been collected during two elections: 17/06/1984 and 10/6/1979.

One can see that the elections’ entropies at the municipality level presents some trends or paths that open different research questions, for example, has found a relationship between the Italian political instability in December 2011–January 2012 and during February 2013 [62], and the financial market; this might be related to the entropy as well. In fact, in our paper, we have found hints about the influence of time, the size of the cities, and so, their economic relevance. This could be investigated in a similar way as it has been done in [41] for the municipality names’ features and their tax contribution between 2007 and 2011.

Talking about the contemporaneous elections, the most interesting part of the analysis to be commented is the one performed over the interception of municipalities across the synchronised elections. Table 3 provides an idea of the overall agreement between the distributions of entropy. These findings have been verified by the usage of the adjusted Gini coefficient, as presented in Section 4.4. The unique cases in which the Kolmogorov–Smirnov tests’ present remarkable hints about similarity in distributions are those reported in Table 3, second sub-table. It is interesting to notice that the Chamber of Deputies and Senate of Republic appear there most of the time and they have the lowest position. These couples are the unique cases in which there are distributional agreements. It is interesting to notice that the ballots of April 2008 and the one of March 2018 have similar electoral laws, made by a proportional division of the seats with a correction carried in by portions of majoritarian. Similar considerations can be done on the cases of June 1897 and June 1983, in fact, they had very similar electoral laws characterized by a proportional allocation of the seats. These political reasons together with further investigations might be at the basis of the recorded changes in entropic behaviours.

Most of the highest statistics in Table 3 come from the results attained involving local elections. This remarks the differences in the selection process made by voters, in fact, as we have discussed in the Introduction, the people tend to use tactical voting behaviours (e.g., ticket-splitting voting) when the candidates can be directly chosen. For example, the elections of April 2008 present a low level of KS test statistics when Chamber of Deputies and Senate of Republic are compared, but, it reports very high values when the outcomes of the Municipal elections are compared against their national respective. The European elections are present in the list of the highest KS tests often paired with the Municipal election. This can be considered as a sign that the way of expressing the preferences for candidates (namely, writing names and surnames as per the Municipal and the European) does not affect the vote distributions that much. From the data we have, we can say this at least for the areas covered by the database for such elections: 12/06/1994, 12/06/2004, 07/06/2009 and 25/05/2014. The entropy distributions have different determinants that change depending on the type of democratic calls or at least on the zones in which the elections are run when the local voters are called. This said, a question remains open here and it is about the north–south division in the country. Different entropic manifestation could be investigated by dividing the country and looking for differences. At this stage, we want to prove the presence of room for such a further investigation.

Between the highest KS statistics of Table 3 there is one pair of national elections (May 2001) that look like an intruder in that list. It is a peculiar case because it presents quite different entropy distributions between the two chambers with respect to the normal regime (see the second part of Table 3). The ballot was characterized by a strongly majoritarian electoral law, which created disagreement because of its complexity. In fact, for that ballot, there was a mechanism called “scorporo” designed to help the smallest parties in getting a seat. The voters could express more than one preference per type and this increased the chaos. For the Chamber of Deputies, the main coalitions: “Casa delle Liberta” and “L’Ulivo” tried to trick the mechanism of “scorporo” creating two fake lists called “liste civetta”. Those lists were supposed to do not get any votes, since they were not advertised to the voters. They obtained preferences anyway, maybe due to the aforementioned complexity/confusion. Furthermore, the 2001 national competition was characterized by a high degree of conflict between the main competitors. Such conflict had spread in the media, capturing the interest of the population, therefore increasing the level of attention to the choices made.

Concluding, the entropic analysis on the votes at the municipality level allows looking at the electoral results from a different perspective, highlighting salient facts and trends in the manifested chaos from the voters’ preference expressions. The specific focus on the concurrent elections by using the Kolmogorov–Smirnov test permits to measure the magnitude of the different disorder realized over the years, it consents to capture differences between National and local ballots dynamics and to capture heterogeneity within the country.

## Figures and Tables

**Table 1:** Italian contemporaneous elections occurred between April 1948 and March 2018. In each column, we have the number of municipality for which elections’ outcomes are present in the dataset, therefore, for them, we have calculated the b-ary entropy.

Election Dates	Municipal	Provincial	Regional	Chamber of Deputies	Senate of the Republic	European
**18/04/1948**				7656	7373	
**07/06/1953**				7847	7858	
**25/05/1958**				7982	7979	
**28/04/1963**				8036	8035	
**19/05/1968**				8054	8054	
**07/05/1972**				8054	8048	
**20/06/1976**				8064	8065	
**03/06/1979**				8079	8074	
**26/06/1983**				8087	8088	
**14/06/1987**				8092	8092	
**05/04/1992**				8100	8101	
**27/03/1994**				8005	7991	
**12/06/1994**	320					8104
**23/04/1995**	5116		6705			
**21/04/1996**				8028	8098	
**13/06/1999**	4492					8098
**16/04/2000**	550		6701			
**13/05/2001**	1265			8001	8099	
**12/06/2004**	4353	5058				8100
**17/04/2005**	1		131			
**03/04/2005**	366	177	6435			
**08/05/2005**	184	377				
**09/04/2006**				8027	8101	
**28/05/2006**	1258	656				
**27/05/2007**	830	647				
**13/04/2008**	423	669		8027	8101	
**07/06/2009**	4094	4591				8100
**28/03/2010**	460	339	6261			
**30/05/2010**	174	377				
**15/05/2011**	1274	732				
**17/11/2013**	2		131			
**24/02/2013**			2058	8018	8092	
**25/05/2014**	3918		1511			8057
**31/05/2015**	679		1456			
**04/03/2018**			1894	7957	7957	

**Table 2:** Correlation between b-ary entropy and adjusted Gini for each election, which fall outside the 5% confidence level on the significance test.

Type of Elections	Dates	N. Obs	ρ	*p*-Value
**Municipal**	1992-06-07	155	0.115	0.153
**Municipal**	1996-11-17	120	0.099	0.280
**Municipal**	1994-11-20	222	0.033	0.627
**Municipal**	1993-11-21	327	−0.030	0.589
**Municipal**	1994-06-12	320	0.029	0.607
**Municipal**	1998-11-29	284	0.052	0.379
**Municipal**	2005-05-08	184	0.124	0.093
**Municipal**	1997-11-16	414	0.014	0.782
**Municipal**	1998-05-24	519	−0.076	0.086
**Municipal**	2000-04-16	550	−0.005	0.914
**Municipal**	1993-06-06	1080	0.054	0.074
**Municipal**	1997-04-27	1110	−0.024	0.428
**Municipal**	2015-05-31	679	−0.039	0.311
**Municipal**	2004-06-12	4353	−0.017	0.249
**Municipal**	2009-06-07	4094	−0.005	0.730

**Table 3:** The highest and the lowest 10 statistics (from the Kolmogorov-Smirnov test) returning from the analysis of municipalities’ entropies recorded for dates of contemporaneous elections when the spatial effect is removed (“interception” of the municipalities). The coupled election types are those listed in “Type 1” and “Type 2”.

Highest KS Statistics					
Dates	Type 1	# municipalities	Type 2	# municipalities	KS statistic
12/06/1994	Municipal	320	European	320	0.894
13/04/2008	Municipal	422	Senate of the Republic	422	0.865
13/04/2008	Municipal	422	Chamber of Deputies	422	0.863
13/05/2001	Municipal	1249	Senate of the Republic	1249	0.850
12/06/2004	Municipal	4342	European	4342	0.803
24/02/2013	Regional	2058	Senate of the Republic	2058	0.802
07/06/2009	Municipal	4093	European	4093	0.785
13/05/2001	Municipal	1241	Chamber of Deputies	1241	0.772
13/05/2001	Chamber of Deputies	7998	Senate of the Republic	7998	0.768
25/05/2014	Municipal	3918	European	3918	0.733
**Lowest KS Statistics**					
Dates	Type 1	# municipalities	Type 2	# municipalities	KS statistic
24/02/2013	Chamber of Deputies	8017	Senate of the Republic	8017	0.206
27/03/1994	Chamber of Deputies	7865	Senate of the Republic	7865	0.157
04/03/2018	Regional	1893	Senate of the Republic	1893	0.151
20/06/1976	Chamber of Deputies	8062	Senate of the Republic	8062	0.148
05/04/1992	Chamber of Deputies	8092	Senate of the Republic	8092	0.138
14/06/1987	Chamber of Deputies	8089	Senate of the Republic	8089	0.094
03/06/1979	Chamber of Deputies	8071	Senate of the Republic	8071	0.076
26/06/1983	Chamber of Deputies	8085	Senate of the Republic	8085	0.074
04/03/2018	Chamber of Deputies	7957	Senate of the Republic	7957	0.071
13/04/2008	Chamber of Deputies	8025	Senate of the Republic	8025	0.028

**Table 4:** The statistics reported in the column “Measures” are based on the b-ary entropy (H columns) and on the adjusted Gini coefficient (G columns) recorded at municipality level for each election type across the years listed in “Dates”.

Dates	Stats	Municipal		Provincial		Regional		Chamber of Deputies		Senate of the Republic		European	
	**Measures**	**H**	**G**	**H**	**G**	**H**	**G**	**H**	**G**	**H**	**G**	**H**	**G**
1948, 1971	N. Obs					6683	6683	39,575	39,575	39,299	39,299		
	Max					0.896	0.974	1.000	1.000	1.000	0.995		
	Min					0.116	0.400	0.000	0.002	0.030	0.002		
	Median *m*					0.654	0.713	0.575	0.787	0.668	0.686		
	Mean μ					0.640	0.712	0.559	0.783	0.655	0.678		
	RMS					0.649	0.717	0.572	0.787	0.667	0.688		
	ST. Dev. σ					0.107	0.083	0.124	0.087	0.128	0.116		
	Var.					0.011	0.007	0.015	0.008	0.016	0.013		
	Sd. Err.					0.001	0.001	0.001	0.000	0.001	0.001		
	Skewness					−0.844	−0.126	−0.681	−1.020	−0.872	−0.645		
	Kurtosis					1.281	0.066	0.932	5.675	1.701	1.828		
	μ/σ					5.971	8.596	4.507	8.980	5.102	5.851		
	3(μ-*m*)/σ					−0.399	−0.051	−0.392	−0.145	−0.319	−0.208		
1972, 1995	N. Obs	7842	7842			33,495	33,495	56,481	56,481	56,459	56,459	32,356	32,356
	Max	1.000	0.987			0.948	0.986	0.941	0.997	1.000	0.999	0.880	0.995
	Min	0.000	0.000			0.096	0.245	0.000	0.000	0.000	0.000	0.035	0.456
	Median *m*	0.927	0.269			0.664	0.716	0.628	0.754	0.662	0.715	0.641	0.749
	Mean μ	0.836	0.276			0.661	0.706	0.626	0.743	0.656	0.699	0.627	0.750
	RMS	0.876	0.335			0.669	0.712	0.637	0.748	0.666	0.708	0.634	0.752
	ST. Dev. σ	0.263	0.189			0.106	0.093	0.115	0.089	0.117	0.114	0.098	0.067
	Var.	0.069	0.036			0.011	0.009	0.013	0.008	0.014	0.013	0.010	0.004
	Sd. Err.	0.003	0.002			0.001	0.001	0.000	0.000	0.000	0.000	0.001	0.000
	Skewness	−2.531	0.298			−0.360	−0.458	−0.675	−0.548	−0.621	−1.320	−1.307	0.108
	Kurtosis	5.267	−0.647			0.220	0.071	1.652	0.561	2.253	3.695	3.658	0.459
	μ/σ	3.174	1.464			6.214	7.553	5.431	8.355	5.600	6.144	6.423	11.220
	3(μ-*m*)/σ	−1.040	0.110			−0.094	−0.312	−0.061	−0.384	−0.150	−0.416	−0.454	0.038
1996, 2018	N. Obs	32,800	32,800	13,623	13,623	28,430	28,430	48,058	48,058	48,455	48,455	32,356	32,356
	Max	1.000	1.000	0.957	0.957	0.950	0.981	0.969	0.984	1.000	0.968	0.930	0.988
	Min	0.000	0.000	0.000	0.272	0.000	0.000	0.000	0.000	0.000	0.000	0.117	0.357
	Median m	0.000	0.286	0.660	0.708	0.718	0.687	0.675	0.736	0.633	0.755	0.682	0.729
	Mean μ	0.312	0.299	0.588	0.703	0.710	0.688	0.677	0.718	0.638	0.737	0.675	0.729
	RMS	0.517	0.368	0.624	0.708	0.715	0.692	0.684	0.724	0.644	0.743	0.679	0.731
	ST. Dev. σ	0.412	0.214	0.210	0.087	0.077	0.077	0.095	0.097	0.085	0.094	0.073	0.057
	Var.	0.169	0.046	0.044	0.008	0.006	0.006	0.009	0.009	0.007	0.009	0.005	0.003
	Sd. Err.	0.002	0.001	0.002	0.001	0.000	0.000	0.000	0.000	0.000	0.000	0.000	0.000
	Skewness	0.683	0.414	−1.105	−0.386	−0.675	−0.185	−0.281	−0.846	0.141	−2.094	−1.559	0.202
	Kurtosis	−1.374	−0.555	0.472	0.304	1.457	0.362	1.015	0.609	2.277	8.717	6.465	1.687
	μ/σ	0.758	1.398	2.793	8.092	9.238	8.954	7.097	7.394	7.524	7.826	9.281	12.852
	3(μ-*m*)/σ	2.274	0.189	−1.030	−0.192	−0.308	0.002	0.063	−0.562	0.180	−0.565	−0.296	−0.038
